# Copper and Zinc as Potential Biomarkers of Mood Disorders and Pandemic Syndrome

**DOI:** 10.3390/molecules27010091

**Published:** 2021-12-24

**Authors:** Magdalena Świądro, Klaudia Ordon, Małgorzata Herman, Dominika Dudek, Renata Wietecha-Posłuszny

**Affiliations:** 1 Laboratory for Forensic Chemistry, Department of Analytical Chemistry, Faculty of Chemistry, Jagiellonian University, 2, Gronostajowa St., 30-387 Krakow, Poland; magda.swiadro@doctoral.uj.edu.pl (M.Ś.); klaudia.ordon@student.uj.edu.pl (K.O.); 2 Group of Toxicological and Pharmaceutical Analyzes, Department of Analytical Chemistry, Faculty of Chemistry, Jagiellonian University, 2, Gronostajowa St., 30-387 Krakow, Poland; malgorzata.herman@uj.edu.pl; 3 Department of Adult Psychiatry, Jagiellonian University Medical College, 21a, Mikołaj Kopernik St., 31-000 Krakow, Poland; dominika.dudek@poczta.fm

**Keywords:** biomarker, copper, zinc, TQ ICP-MS, mood disorders

## Abstract

The diagnosis of affective disorders has been the subject of constant research by clinicians from all over the world for many years. Making an appropriate diagnosis among patients suffering from mood disorders is sometimes problematic due to the personality-changing nature of patients and the similarity in the clinical picture of episodes in affective disorders. For this reason, there is a need to develop rapid and effective methods of determining biological markers that differentiate these diseases. The research was carried out with blood taken from 15 patients and 15 volunteers. The analysis of biological material for trace concentrations of zinc and copper was carried out with the use of ultrasensitive triple-quadrupole inductively coupled plasma mass spectrometry (TQ ICP-MS). The obtained results prove that the concentration of copper in the test group was lower than in the control group. For the zinc concentrations, the inverse relationship was observed. The group of patients was characterized by a higher concentration of this element than the group of healthy volunteers. Summarizing the obtained results and comparing them with the results of studies by other authors, it was found that zinc and copper may be potential biomarkers of affective disorders and pandemic syndrome.

## 1. Introduction

Mood disorders are not only widely understood depressive syndromes, but also bipolar spectrum disorders. Due to their increasing prevalence, people are also becoming increasingly aware of the existence and differentiation of uni-and bipolar affective disorders [1]. Bipolar disorder (BD) is a complex neuropsychiatric disorder. It is characterized by early onset, very high relapse, and heredity [2]. Major depressive disorder (MDD) is also called recurrent depressive disorder or defined as a single depressive episode. Patients with pre-disease MDD often exhibit melancholic and introverted personality traits [3].

Depressive disorders have a complex etiology in which the interaction of susceptibility and environmental factors, especially those related to stress, plays a crucial role. The United Nations [4] and WHO [5] are alerting that the COVID-19 pandemic and the stress related to it will contribute to an increase in anxiety and depressive disorders [6]. Ettman et al. [7] conducted a study on 1441 respondents from the COVID-19 pandemic and 5065 respondents from before the pandemic. Stress related to fear of infection, concerns for the health of the family, forced isolation and loneliness, economic problems, such as the threat of job and income loss, the specter of the economic crisis, and the inability to plan the future—all reactive factors—meant that the prevalence of depression symptoms was more than three-fold higher during the COVID-19 pandemic than before [7].

Since the current diagnosis of mood disorders is based only on the patient’s history and clinical picture analysis, this contributes to a biased diagnosis of the disease. Of all mental disorders, BD is most often confused with MDD because, during bipolar disorder, the first symptom is most often a depressive episode [2]. Due to the fact that one of the many side effects of the COVID-19 pandemic will be the soaring number of people suffering from mood disorders, it is important to effectively differentiate between affective disorders. Therefore, there is an urgent need to develop methods for the assay of biological markers differentiating between uni-and bipolar affective disorder. Due to the fact that the diagnosis will be based on the results of laboratory tests, it will become unambiguous and beyond doubt.

Copper (Cu) and zinc (Zn) are among the 11 trace elements meaningful to human health [8]. Both of these microelements participate in many physiological processes that are crucial in the etiology of mental disorders. These processes include, among others: NMDA (N-methyl-D-aspartate) receptor action, regeneration, and repair of the nervous system—brain-derived neurotrophic factor (BDNF), nerve growth factor (NGF), and copper are part of ceruloplasmin, which is involved in the processes underlying affective disorders [9].

Until now, few studies have been conducted on serum copper levels in individuals suffering from unipolar and bipolar disorder. The results are inconclusive, but, in most of them, you can find confirmation that, during the depressive episode, patients have significantly increased copper levels in their blood. One of the solid foundations of this assumption are the studies carried out by Manser et al. [10], which showed that the blood serum samples of depressed patients had an elevated concentration of ceruloplasmin, which is the main transporter of copper in the body.

Research into the role of zinc in mood disorders began in 1983 when a case was described linking zinc deficiency with recurrent depression disorders [11]. It is well known that Zn also has antiviral properties, supports immunity, and improves the weakened function of immune cells [12]. It turned out that, in times of a global pandemic, when people are looking for fast, safe, cheap, accessible, and effective solutions, zinc supplementation can also be helpful in the fight against the SARS-CoV-2 virus or its effects in the body [12,13,14]. The conducted research has shown that the facilitator of infection with SARS-CoV-2 virus is the activity of the ACE-2 enzyme, the expression of which is determined by the Sirt-1 gene. Since zinc reduces the activity of the Sirt-1 gene, it is anticipated that it may reduce ACE-2 expression and, thus, virus entry into the cell [14]. In this difficult and, above all, unexpected situation for the world, special attention should be paid to zinc, the supplementation of which brings benefits both in the fight against the SARS-CoV-2 virus itself and in counteracting the side effects of the COVID-19 pandemic—supporting psychopharmacotherapy.

The research carried out is a preliminary study aimed to determine the trace amounts of copper and zinc in the whole blood of patients suffering from MDD and BD and to compare the results obtained with blood samples taken from volunteers of the so-called ‘control group’. The analysis of biological materials was carried out using an innovative method of triple-quadrupole inductively coupled plasma mass spectrometry (TQ ICP-MS).

## 2. Results and Discussion

### 2.1. General Presentation of the Results Obtained

The population of the study was made up of 15 affective disorder patients and 15 healthy control subjects. Age, sex, and information regarding BD or MMD phase, cigarette smoking, and drinking alcohol were compared. The summarized copper concentration in the patients’ blood was in the range of 0.88 ± 0.13 mg/L but, in the blood collected from the control group, was in the range of 1.05 ± 0.32 mg/L. In the case of zinc, the concentrations were in the range of 4.97 ± 1.79 mg/L in the patients’ blood and in the range of 3.75 ± 0.69 mg/L in the control group. Table 1 presents the concentration of biomarkers results of each of the analyzed criterion of the collected blood samples of the studied groups.

The results of the research carried out so far on the summarized concentration of copper in the blood of people suffering from mood disorders are inconclusive. The results obtained in this study are consistent with some of the studies published by other authors. For example, Siwek et al. [15] cited a study on a group of patients before and after pharmacotherapy, where the authors obtained the mean concentration of copper in the blood of patients before starting treatment, amounting to 1.18 mg/L, but after pharmacotherapy, 1.04 mg/L. In addition, in a group of healthy volunteers, the average level of copper concentration was 1.19 mg/L. In the described study, the authors did not analyze the concentration of micronutrients before and after the end of treatment, although the studied group of patients was on drugs. The results obtained are, therefore, partly in line with the results obtained in the presented publication. The control group has a higher blood copper concentration than the drug-treated group.

In another study, Maes et al. [9] suggested that serum levels of ceruloplasmin increased in patients suffering from mood disorders. It is related to the acute phase response seen in depression. Therefore, a reduction in blood copper concentration in patients undergoing pharmacotherapy may indicate satisfactory resulting treatment.

Different results from the above were obtained by Masner et al. [10]. In their study, the mean concentration of copper in the diagnosed patients was 1.14 ± 0.23 mg/L, while in the control group it was 0.94 ± 0.11 mg/L. It is worth noting, however, that they conducted research on a group of patients who did not undergo pharmacotherapy. This may be one of the important reasons for obtaining the results showing the opposite relationship to this study: 0.88 ± 0.13 mg/L for the test group and 1.05 ± 0.32 mg/L for the control group. It is also satisfactory that the concentrations of copper in the blood of patients and healthy people were within the generally accepted standards, which are 0.75–1.5 mg/L [16].

Disturbing is the fact that the concentrations of zinc in the study groups differed significantly from the adopted standards (0.7–1.2 mg/L) [16]. However, as is commonly known, zinc is an element from which many alloys used in everyday objects are made, including door handles or scissors. Moreover, the surprisingly high concentration of this element occurs in the air, as well as in house dust. Rasmussen et al. [17] conducted a study of house dust on the content of metals such as copper, zinc, and nickel. The study showed a very high average concentration of zinc in the dust, which was 793 μg/g. Due to the high presence of zinc in the biosphere, the probability of contamination of samples during the analysis is highly increased. In addition, Lu et al. [18] researched differences in direct dilution of blood as an alternative to acid digestion for the improvement of analytical productivity when measuring trace elements using inductively coupled plasma mass spectrometry (ICP-MS). This study compared these two sample preparation methods for the ICP-MS determination of multiple elements in human blood and serum. In addition, it was analyzed in whole blood, the same as in the described research. In the case of Zn, a high concentration was also obtained—acid digestion: 5.6 ± 0.61 mg/L and alkali dilution: 6.2 ± 0.64 mg/L. Moreover, the authors analyzed reference materials where each result was in the acceptable range [18].

The behavior of many of the discussed analogies in the concentrations of zinc between the studied groups, in comparison with the results of studies by other authors, gives hope that the conducted analysis, despite the excessive concentration of zinc, shows the correct relationships.

### 2.2. Biomarkers Level by Gender

The analysis of copper concentrations, taking into account the division by gender, showed no significant differences between the studied groups.

**Figure 1:**
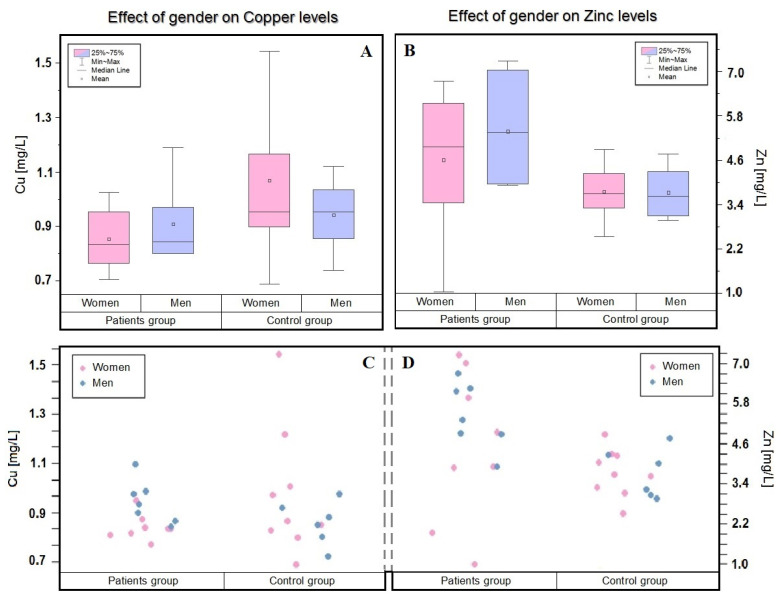
Dependence of the concentration of zinc and copper in the blood of patients and the control group depending on the selected criterion—gender (In patient group: women—pink; *n* = 8, men—blue; *n* = 7. In control group women—pink; *n* = 9, men—blue; *n* = 6). The mean (circle), median (line), percentile 25, 75 (boxes), and minimum and maximum value (whiskers) are shown in (**A**,**B**). (**A**) Box plot showing the influence of gender on the concentration of copper. (**B**) Box plot showing the influence of gender on the concentration of zinc. The visualizations in (**C**,**D**) were inspired by the *t*-test, i.e., they are an attempt to compare the means of two finite research samples with selected criteria. (**C**) Shows visualizations of the distribution of copper concentrations considering the gender criterion. (**D**) Shows visualizations of the distribution of zinc concentrations considering the gender criterion.

As can be seen in Table 1 and Figure 1, in the studied group of patients, women are characterized by a much lower concentration of this element. In the men of both the control and patient groups, the copper level is around 0.9 mg/L. Interesting observations, which are consistent with our results, were made by K. Styczeń et al. [19] in their study, from which 114 (86 women and 28 men) patients and 50 (36 women and 14 men) controls were selected. Significantly, no differences between men from the control and patient groups and, moreover, lower copper levels in healthy men than in healthy women were observed.

In the presented research, was analyzed a group contained women (53.3%) and men (47.7%). The women, the group observed a decrease in Zn concentration, which could not be observed in a group of men. This observation is also supported by the works of other authors. A cross-sectional study by Masereijan et al. [20] also showed that women are characterized by a lower concentration of Zn in the blood and that they are more likely to experience depressive symptoms.

### 2.3. Biomarkers Level Depending on the Type of Disease

The main aim of the presented study was to determine the trace amounts of copper and zinc in the blood of patients with an affective disorder. As a result, the mean level of copper concentration in the group of patients was lower than in the group of healthy volunteers.

In the patients’ blood it was in its range of 0.88 ± 0.13 mg/L but, in the blood collected from the control group, it was in the range of 1.05 ± 0.32 mg/L. Moreover, lower levels of copper were observed in patients diagnosed with bipolar disorder than in patients suffering from depression. In the blood of patients with a diagnosis of BD, the mean concentrations of copper were 0.84 ± 0.04 mg/L, while in the control group: 1.05 ± 0.32 mg/L.

The conducted study showed an increased concentration of copper in patients suffering from unipolar affective disorder compared to patients with bipolar disorder. This result was also confirmed in the study by Styczeń et al. [21]. In another publication by Siwek et al. [22], the authors showed that patients with bipolar disorder have lower blood Cu levels than the control group. These results are consistent with those presented in this paper.

**Figure 2:**
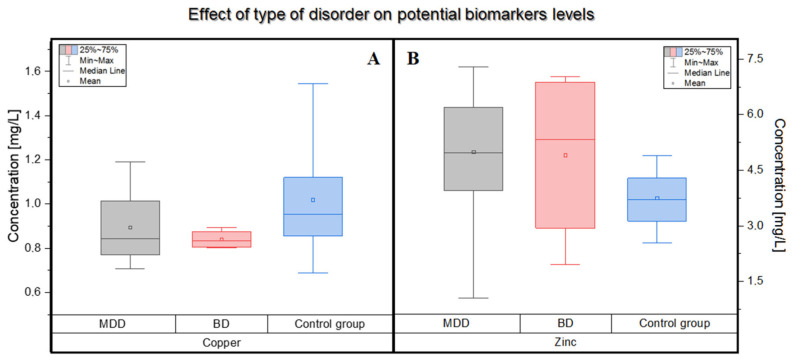
Differences in the concentration of copper and zinc in the blood of patients suffering from BD (pink, *n* = 4) and MDD (grey, *n* = 11) compared to the control group (blue, *n* = 15). The mean (circle), median (line), percentile 25, 75 (boxes), and minimum and maximum value (whiskers) are shown in both figures. (**A**) Box plot showing the influence of type of disease or no disease on the concentration of copper. (**B**) Box plot showing the influence of the type of disease or no disease on the concentration of zinc.

A different situation occurs when comparing zinc concentrations in the studied groups. Contrary to the hypothesis, the level of Zn in the blood of patients turned out to be significantly higher than in the healthy control group (Figure 2). In a study conducted by Siwek et al. [23], zinc concentrations were measured in patients diagnosed with bipolar disorder, taking into account its various types, and the obtained result was compared with the control group. The authors showed an increased concentration of Zn in patients with bipolar disorder in the current manic or hypomanic episode compared to the control group. In this study, patients were not distinguished according to the types of bipolar disorder, but the mean concentration of zinc in their blood also turned out to be higher than in the control group. González-Estecha et al. [24] also observed such a relationship. According to Siwek et al. [23], the manic or hypomanic state may be differentiated from the state of depression by the concentration of zinc. The authors of this study also indicated many other substances, such as dopamine or norepinephrine, the levels of which vary depending on the patient’s medical condition.

### 2.4. Effect of Smoking on Biomarkers Levels

**Figure 3:**
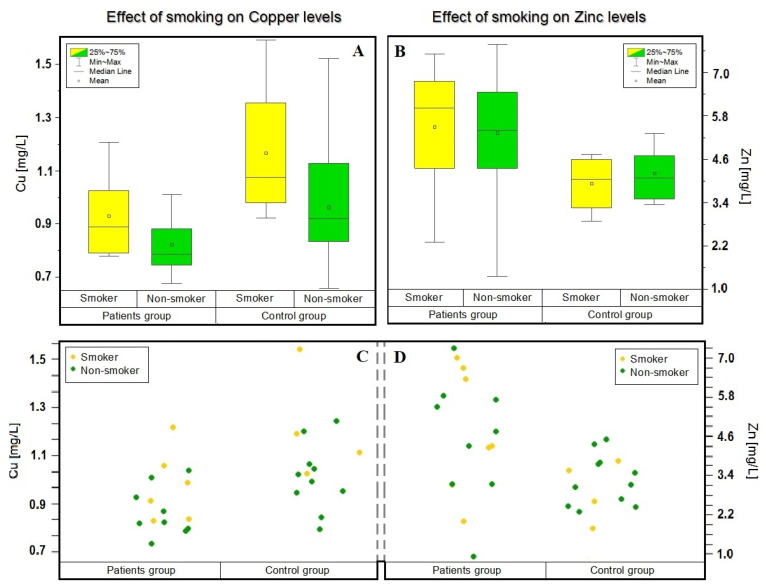
Dependence of the concentration of zinc and copper in the blood of patients and the control group depending on the selected criterion—smoking (In patient group: smoker—yellow; *n* = 6, non-smoker—green; *n* = 9. In control group: smoker—yellow; *n* = 4, non-smoker—green; *n* = 11.). The mean (circle), median (line), percentile 25, 75 (boxes), and minimum and maximum value (whiskers) are shown in (**A**,**B**). (**A**) Box plot showing the influence of smoking on the concentration of copper. (**B**) Box plot showing the influence of smoking on the concentration of zinc. The visualizations in (**C**,**D**) were inspired by the *t*-test, i.e., they are an attempt to compare the means of two finite research samples with selected criteria. (**C**) Shows visualizations of the distribution of copper concentrations considering the smoking criterion. (**D**) Shows visualizations of the distribution of zinc concentrations considering the smoking criterion.

Considering the criterion of smoking, an interesting effect of smoking on the level of copper concentration was observed. In the blood of both analyzed groups, people declaring smoking were characterized by a higher concentration of this element. In the control group—smoking: 1.26 ± 0.48 [mg/L] and non-smoking: 0.97 ± 0.22 [mg/L]—and in the patients—smoking: 0.94 ± 0.15 [mg/L] and non-smoking: 0.84 ± 0.10 [mg/L]. Authors of other studies made similar observations, e.g., the team of Li [25] and Siwek [22]. Moreover, the concentration of copper was higher in people declaring habitual smoking in each group. Results are shown in Figure 3.

## 3. Materials and Methods

### 3.1. Subject

The present study includes 15 patients (7 men and 8 women; mean age: 54 years) hospitalized in the Department of Adult Psychiatry at the Jagiellonian University Medical College. In this group, we diagnosed 4 patients with bipolar disorder (including the diagnostic subtypes according to the DSM-IV classification) and 11 with depression. Three patients were also diagnosed with other mood disorders, such as schizophrenia, anxiety, and depression disorders, and specific personality disorders. Healthy controls included 15 volunteers without bipolar disorder or depression (6 men and 9 women; mean age: 41 years). Patients participating in the study were treated with standard medications used in the treatment of mood disorders such as sertraline, trazodone, etc. Both in the control and patient group, there was no person who additionally took preparations containing copper or zinc.

For both patients and the control group, demographic and clinical data, including age, sex, and information regarding depressive or manic/hypomanic phase, drugs applied in treatment, cigarette smoking, alcohol, and history of substance abuse, were obtained. Patients and controls gave their informed written consent to the study and the study was conducted according to the guidelines of the Declaration of Helsinki and approved by the Bioethics Committee (approval no. 1072.6120.302.2018). In addition, according to the study protocol, from each patient and healthy volunteer, no more than 2.4 mL of venous blood was obtained. All samples were stored at a temperature of −20 °C prior to the analysis, in accordance with standard laboratory practice.

### 3.2. Chemical and Reagents

Concentrated nitric acid (65%) was purchased from Avantor Performance Materials POCH (Gliwice, Poland). Ultrapure water (18.2 MΩ cm, <3 ppb TOC), which was used to prepare all aqueous solutions, was generated with the Milli-Q system by Merck (Darmstadt, Germany). ICP multielement XVI pattern was obtained from Merck (Darmstadt, Germany).

### 3.3. Sample Preparation

**Figure 4:**
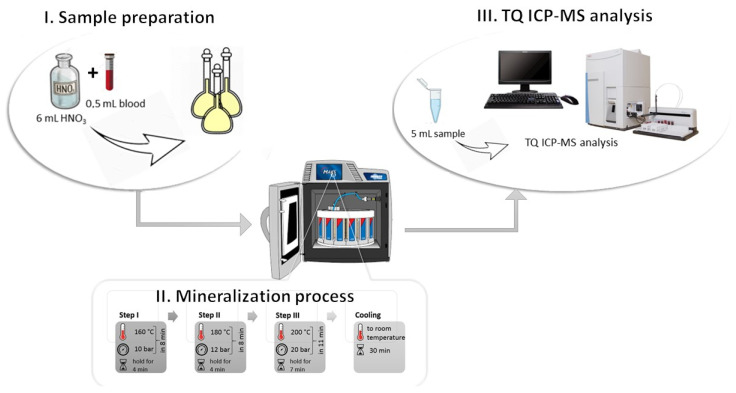
Sample preparation methodology divided into three main sections with included detail on the stage of the mineralization process—temperature and pressure. I. Sample preparation: add 6 mL concentrated HNO_3_ to 0.5 mL of blood and transfer in a special microwave vessel applied for mineralization—wait 30 min. II. Mineralization process: the three-step microwave program. III. TQ ICP-MS analysis: preparation of 5 mL of mineralizate for analysis.

In the research for elemental analysis, the samples were used with a microwave digestion system Mars 5X (Matthews, NC, USA). First, 0.5 mL of blood was transferred in a special microwave vessel applied for mineralization. Then, 6 mL of concentrated HNO_3_ was added for each probe. The vessels were covered with a watch glass and left for reaction initiation at room temperature for 20–30 min. After that, the three-step microwave program was used for the decomposition of samples: I: ramp to 160 °C (pressure 10 bar) in 8 min and hold for 4 min; II: ramp to 180 °C (pressure 12 bar) in 8 min and hold for 4 min; III: ramp to 200 °C (pressure 20 bar) in 11 min and hold for 7 min. Average furnace power throughout the microwave oven process was 800 W. After cooling to room temperature (30 min), the digests were diluted to 10 mL with deionized water. The prepared samples were stored at 4 °C and analyzed using TQ ICP-MS. The sample preparation procedure is presented in Figure 4.

### 3.4. TQ ICP-MS Method

Each sample was measured in triplicate. All measurements were carried out with the triple-quadrupole inductively coupled plasma mass spectrometer (TQ ICP-MS) (Thermo Fisher Scientific, Bremen, Germany) equipped with auto-sampler ASX-560 (Teledyne CETAC Technologies, Omaha, NE, USA). The instrument was tuned daily for the highest sensitivity. The typical parameters for copper and zinc analysis are shown in Table 2.

### 3.5. Data Analysis

Copper and zinc levels were presented as the standard deviation for the mean value (mean ± SD) and compared between the patients’ case and control groups. The visualizations of the results were made using the RStudio software. The visualizations were inspired by the *t*-test, i.e., they are an attempt to compare the means of two finite research samples with selected criteria. The idea behind the construction of the visualization was to present the values for the group in a way that allows for easy interpretation of the relationship between individual measurements or averages. To make it possible, the points were placed on one plane and dispersed using a random function. Due to this, they are arranged in space and not grouped in one line.

## 4. Conclusions

The diagnosis of affective disorders remains a considerable challenge for physicians and diagnosticians due to the similarities in the clinical picture of depressive episodes in affective disorders. Moreover, a significant increase in the number of cases of mood disorders is observed every year and, considering the side effects of the COVID-19 pandemic, we must be prepared for a greater increase in the number of people suffering from mood disorders than anticipated so far. For this reason, it is essential to develop methods for marking potential biomarkers that are effective in differentiating these diseases. The described research includes analyzing the levels of potential biomarkers, such as copper and zinc, that are important in the etiology of mood disorders. The analysis of biological materials was carried out using the most modern and ultrasensitive technique of mass spectrometry coupled with inductively excited plasma with a triple ion analyzer (TQ ICP-MS). Summarizing the obtained results and taking into account the limitations of this study, a decreased concentration of copper in the blood of the studied group was found, which is contrary to the assumed hypothesis. Perhaps this result was significantly influenced by the fact that the studied group of patients was subjected to pharmacotherapy, which contributed to the normalization of the copper concentration in their body.

Despite the overestimated concentration of zinc in the tested samples, the differences in concentrations in individual groups maintained analogies supported by the results of studies by other authors. Higher levels of zinc were observed in patients with bipolar disorder than in the control group. However, similar studies conducted should be divided into different types of bipolar disorder, according to Siwek et al. [23], as patients with the current manic/hypomanic episode are characterized by elevated levels of zinc.

It is worth emphasizing that the conducted study is a preliminary study using the innovative TQ ICP-MS technique, which has not yet been used in research on potential biomarkers of affective disorders. The results obtained in this study suggest the need to confirm them in future research with an increased size of the groups and with the division into additional criteria. Perhaps the use of the innovative TQ ICP-MS technique for this type of analysis will help to explain the importance of copper, zinc, and other microelements during affective disorders.

## Figures and Tables

**Table 1:** Zn and Cu values in blood serum [mg/L] in control and patient groups. The results were analyzed according to criteria: gender, smoking, type of disease. Results are presented as mean ± standard deviation.

Criterion		Mean ± SD [mg/L]			
		*n*	Patients	*n*	Controls
**Copper**					
Total level		15	0.88 ± 0.13	15	1.02 ± 0.24
Gender	Women	8	0.86 ± 0.12	9	1.07 ± 0.29
	Men	7	0.91 ± 0.14	6	0.94 ± 0.13
Tobacco	Smoker	6	0.94 ± 0.15	4	1.16 ± 0.27
	Non-smoker	9	0.84 ± 0.10	11	0.97 ± 0.22
Type of disease	BD	4	0.84 ± 0.04	- *	- *
	MDD	11	0.89 ± 0.15	- *	- *
**Zinc**					
Total level		15	4.97 ± 1.79	15	3.75 ± 0.69
Gender	Women	8	4.61 ± 2.05	9	3.76 ± 0.71
	Men	7	5.38 ± 1.49	6	3.73 ± 0.74
Tobacco	Smoker	6	5.07 ± 1.87	4	3.54 ± 0.81
	Non-smoker	9	4.91 ± 1.86	11	3.82 ± 0.67
Type of disease	BD	4	4.91 ± 2.42	- *	- *
	MDD	11	4.99 ± 1.65	- *	- *

* No data are available as only patients were classified by disease type.

**Table 2:** Operating conditions of iCAP TQ ICP-MS.

Parameter	Value
Nebulizer	MicroMist Nebulizer 0.2 mL∙min^−1^
Spraychamber	Quartz cyclonic spraychamber cooled to 2.6 °C
Nebulizer Gas Flow	1 L∙min^−1^
RF Power	1548.5 W
SQ-KED Gas Flow	100% He, 0.8 L∙min^−1^
Dwell time	0.1 s
Q1 resolution Cu	Normal

## Data Availability

Not applicable.

## Sample Availability

Samples of the compounds are not available from the authors.
